# Novel bioemulsifier produced by a *Paenibacillus* strain isolated from crude oil

**DOI:** 10.1186/s12934-015-0197-5

**Published:** 2015-01-31

**Authors:** Eduardo J Gudiña, Jorge FB Pereira, Rita Costa, Dmitry V Evtuguin, João AP Coutinho, José A Teixeira, Lígia R Rodrigues

**Affiliations:** CEB – Centre of Biological Engineering, University of Minho, 4710-057 Braga, Portugal; CICECO – Chemistry Department, University of Aveiro, 3830-103 Aveiro, Portugal

**Keywords:** Surface active compound, Bioemulsifier, *Paenibacillus* sp, Bioremediation

## Abstract

**Background:**

Surface active compounds produced by microorganisms are attracting a pronounced interest due to their potential advantages over their synthetic counterparts, and to the fact that they could replace some of the synthetics in many environmental and industrial applications.

**Results:**

Bioemulsifier production by a *Paenibacillus* sp. strain isolated from crude oil was studied. The bioemulsifier was produced using sucrose with and without adding hydrocarbons (paraffin or crude oil) under aerobic and anaerobic conditions at 40°C. It formed stable emulsions with several hydrocarbons and its emulsifying ability was not affected by exposure to high salinities (up to 300 g/l), high temperatures (100°C-121°C) or a wide range of pH values (2–13). In addition, it presented low toxicity and high biodegradability when compared with chemical surfactants. A preliminary chemical characterization by Fourier Transform Infrared Spectroscopy (FT-IR), proton and carbon nuclear magnetic resonance (^1^H NMR and ^13^C CP-MAS NMR) and size exclusion chromatography indicated that the bioemulsifier is a low molecular weight oligosaccharide-lipid complex.

**Conclusion:**

The production of a low molecular weight bioemulsifier by a novel *Paenibacillus* strain isolated from crude oil was reported. To the best of our knowledge, bioemulsifier production by *Paenibacillus* strains has not been previously reported. The features of this novel bioemulsifier make it an interesting biotechnological product for many environmental and industrial applications.

**Graphical Abstract:**
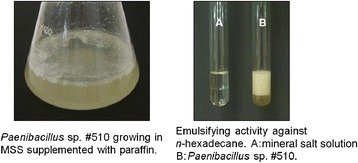
Novel bioemulsifier from *Paenibacillus* sp.

## Background

Microbial surface-active compounds (SACs) are amphiphilic metabolites consisting of hydrophobic and hydrophilic moieties which allow them to partition at the interface between fluid phases with different degrees of polarity. These compounds comprise a wide variety of structures, and can be divided into two main groups: low molecular weight and high molecular weight SACs. Low molecular weight molecules reduce surface and interfacial tension as their primary activity. They include lipopeptides and glycolipids among others, and their molecular masses range from 500 to 1500 Da [1]. On the other hand, high molecular weight polymers, so-called bioemulsifiers, are more effective in forming and stabilizing oil in water or water in oil emulsions, but do not necessarily reduce surface or interfacial tension. Usually they consist of polymeric structures including polysaccharides, proteins, lipids and complexes of these molecules. Although less studied than biosurfactants, bioemulsifiers production has also been described in different bacteria [2-11], yeasts [12-15] and filamentous fungi [16]. Bioemulsifiers exhibit similar or better emulsifying activities when compared with their chemical counterparts, and because of their biological origin, they are considered less toxic and more easily biodegradable, thus implying a greater environmental compatibility [8,17,18].

Bioemulsifiers, due to their properties, could replace some of the chemically synthesized emulsifiers in bioremediation, enhanced oil recovery, clean-up of oil contaminated pipes and vessels, as additives in cleaning products and laundry formulations, and as emulsion-stabilizing agents in the food, cosmetic or pharmaceutical industries [5,6,8,10,13,15,16,19,20]. For instance, numerous patents have been issued claiming the application of bioemulsifiers produced by members of the genus *Acinetobacter* in bioremediation, in the petroleum industry, or as additives in personal care products, paints, dyes and cosmetics, among others (reviewed by Shete et al. [21]).

Despite their potential applications, the large-scale production of bioemulsifiers has not been achieved due to their high production costs. While high production costs can be tolerated for compounds used at low concentrations in highly-priced products, such as cosmetics, they are unaffordable for applications that require high volumes of low-priced compounds. One of the advantages of bioemulsifiers when compared with the synthetic emulsifiers, for applications such as bioremediation, is that they can be produced *in situ* by selected microorganisms using inexpensive substrates, which constitutes a cheaper option than the addition of a purified compound. The development of new and cheaper processes, the use of low-cost raw materials and the isolation of new microorganisms capable of producing bioemulsifiers in an efficient manner can reduce their production costs and increase their competitiveness [1,22].

The aim of this work was to study the bioemulsifier production by a new isolate obtained from crude oil. The preliminary chemical structure of the bioemulsifier and its functional properties were studied. Furthermore, its toxicity and degradability were evaluated.

## Results and discussion

### Characterization of the bioemulsifier-producing strain

**Figure 1:**
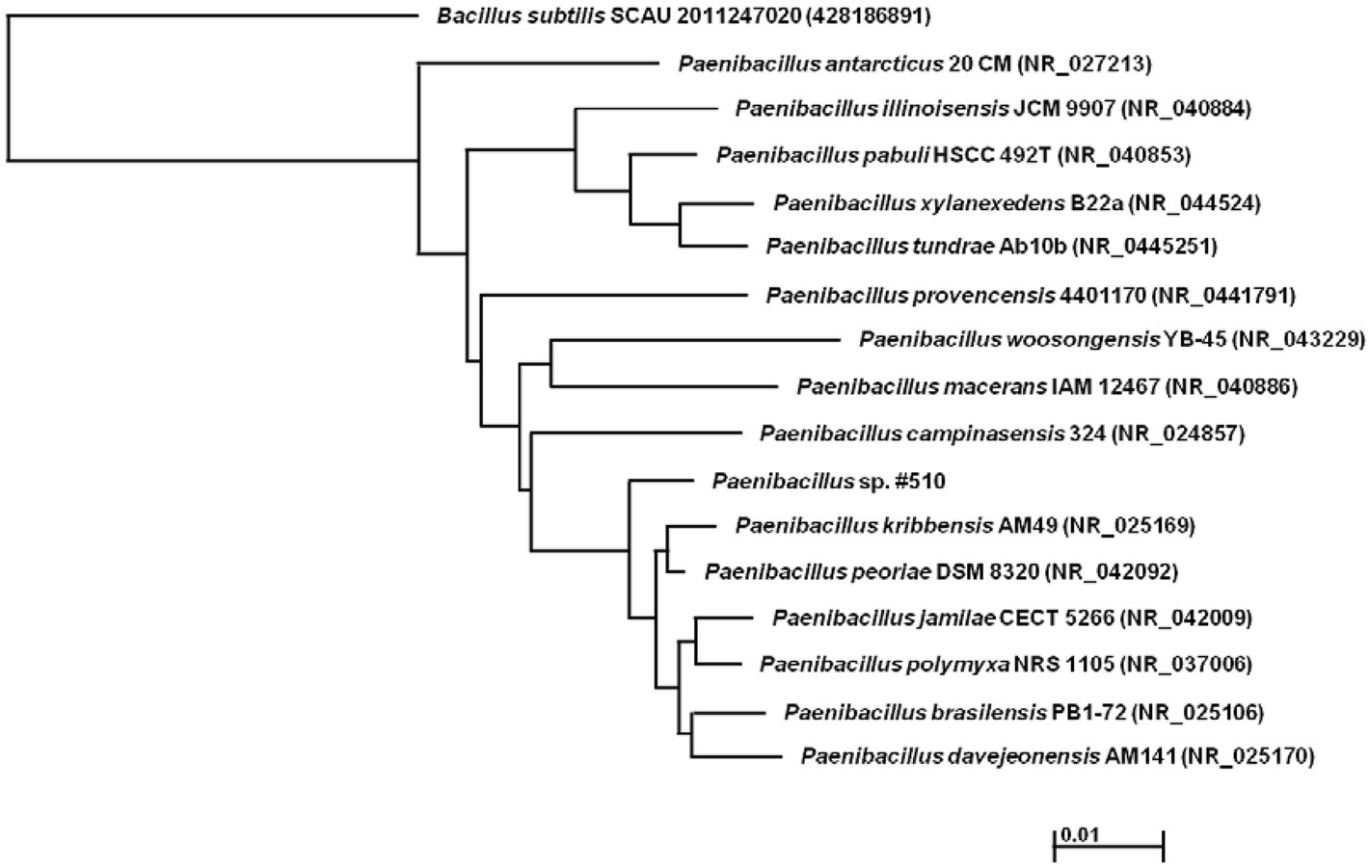
**Phylogenetic neighbor-joining tree based on the 16S rRNA sequence of** ***Paenibacillus*** **sp. #510.**

The isolate #510 was identified according to the partial sequence obtained from its 16S rRNA gene. The 1451 bp sequence obtained was compared with those deposited at the NCBI database, and revealed the highest similarity (99%) with different species belonging to the genus *Paenibacillus* (Figure 1). According to the results, the isolate was designated as *Paenibacillus* sp. #510. The partial sequence of the 16S rRNA gene was deposited in the GenBank database under accession number KF151179. The strain was deposited on Micoteca da Universidade do Minho (MUM) culture collection under the reference number MUM 14.03.

### Effect of aeration and hydrocarbons on bioemulsifier production

*Paenibacillus* sp. #510 was found to produce an extracellular emulsifying agent (bioemulsifier). The bioemulsifier production was studied using MSS medium (with and without sucrose) supplemented with paraffin (5% w/v) or crude oil (5% w/v) under aerobic and anaerobic conditions. The results obtained are shown in Table 1.

**Table 1:** **Effect of aeration and hydrocarbons on bioemulsifier production**

**Medium**	**ST (mN/m)**	**E**_**24**_ **(%)**	**[Emulsifier] (g/l)**	**[Biomass] (g/l)**
**Anaerobic (120 rpm)**				
**MSS**	52.9 ± 1.5	36.4 ± 4.4	5.0 ± 0.5	0.437 ± 0.086
**MSS + Paraffin**	51.1 ± 1.8	43.1 ± 3.1	5.6 ± 0.2	0.523 ± 0.043
**MSS + Crude Oil**	50.6 ± 1.4	42.1 ± 2.1	5.4 ± 0.4	0.496 ± 0.091
**Aerobic (120 rpm)**				
**MSS**	51.1 ± 0.9	49.9 ± 2.7	6.1 ± 0.2	0.846 ± 0.112
**MSS + Paraffin**	50.1 ± 1.6	64.4 ± 1.6	7.9 ± 0.1	0.904 ± 0.099
**MSS + Crude Oil**	50.0 ± 0.6	62.1 ± 2.5	7.4 ± 0.3	0.885 ± 0.121

Surface tension values (mN/m), emulsifying activity (E_24_, %) and biomass and bioemulsifier concentrations (g/l) obtained with *Paenibacillus* sp. #510 grown at 40°C under aerobic (7 days) and anaerobic (10 days) conditions in MSS and MSS supplemented with paraffin (5% w/v) or crude oil (5% w/v). The emulsifying indexes were performed using *n*-hexadecane. The surface tension and the emulsifying activity of MSS medium were 70.3 ± 0.4 mN/m and 0.0%, respectively. Results represent the average of three independent experiments  ±  standard deviation.

The use of paraffin or crude oil as sole carbon sources yielded no growth. However, the addition of paraffin or crude oil to the culture medium containing sucrose resulted in emulsification values higher than those obtained without hydrocarbons, both under aerobic and anaerobic conditions (Table 1). Therefore, both hydrocarbons induced the bioemulsifier production by *Paenibacillus* sp. #510. The best emulsifying activity against *n*-hexadecane (64.4 ± 1.6%) was obtained when *Paenibacillus* sp. #510 was grown under aerobic conditions and in the presence of paraffin. This activity was in accordance with data reported for other bioemulsifier-producing microorganisms [8,9,13,20,23]. Furthermore, the emulsions formed remained stable for one month at 40°C.

The amount of bioemulsifier produced by *Paenibacillus* sp. #510 under aerobic conditions (6.1-7.9 g/l) was similar to those reported for yeasts and *Streptomyces* species [13,14,20], but considerably higher when compared with the amounts produced by other bacteria (between 1.4 and 4.2 g/l) [3,5,9,11]. Moreover, the bioemulsifier produced by *Paenibacillus* sp. #510 did not significantly reduce the surface tension of the culture medium, which is the general characteristic of bioemulsifiers produced by a number of bacteria, yeasts and filamentous fungi [4,6,8-12].

To our knowledge, bioemulsifier production by *Paenibacillus* strains has not been previously reported. Najafi et al. [24] isolated a *Paenibacillus alvei* strain from an Iranian oil field that produced a lipopeptide biosurfactant able to reduce the surface tension up to 35 mN/m. However, no emulsifying activity was reported by the authors for that isolate.

### Kinetics of bioemulsifier production

**Figure 2:**
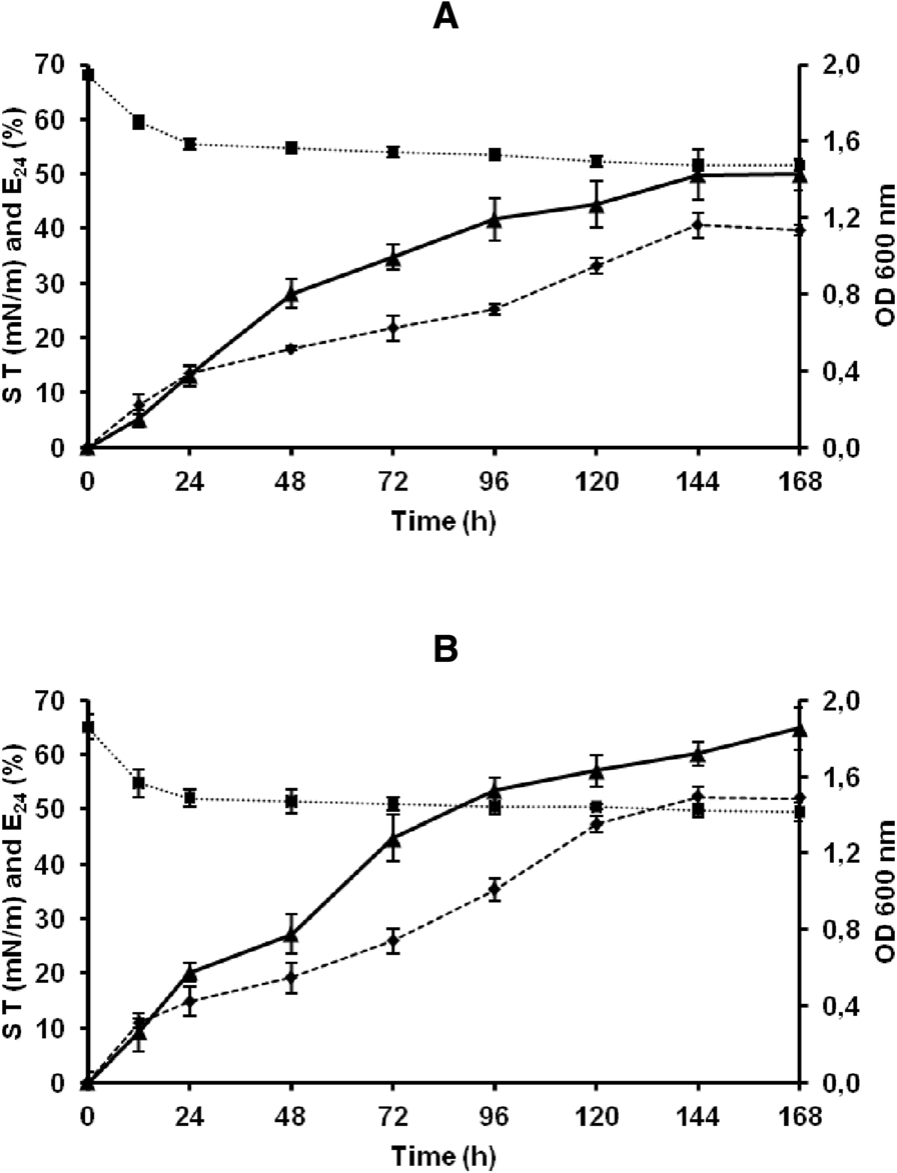
**Kinetics of bioemulsifier production.** Time course profiles of cell growth (♦), surface tension (■) and emulsifying activity (▲) of *Paenibacillus* sp. #510 grown in MSS medium **(A)** and MSS medium supplemented with paraffin (5% w/v) **(B)** at 40°C and 120 rpm under aerobic conditions. Results represent the average of three independent experiments.

Figure 2 shows the kinetics of cell growth and bioemulsifier production by *Paenibacillus* sp. #510 in MSS and MSS medium supplemented with paraffin under aerobic conditions. Bioemulsifier production was found to be growth-associated in both cases, as a parallel relationship was observed between the biomass production and the emulsifying activity. The same profile was observed for several other bioemulsifier-producing microorganisms [7,8,20,23]. However, in other reports, a partially growth-associated bioemulsifier production profile was observed, in which the bioemulsifier production continued during the stationary growth phase [12]. In the current study, cell growth and bioemulsifier production were higher in medium supplemented with paraffin. The highest emulsifying activity was achieved at 168 hours in both media. After that point, the emulsifying activity decreased probably due to the degradation of the bioemulsifier.

### Effect of salinity, pH and temperature on emulsifier stability

The applicability of SACs in several fields depends on their stability at different temperatures, salinities and pH values. In order to assess the effect of salinity on the emulsifier activity, bioemulsifier solutions (1 g/l) were supplemented with different NaCl concentrations, ranging from 10 to 300 g/l, and then emulsifying activity was measured. The addition of NaCl, even in amounts above the limit of saturation (300 g/l), did not have a negative effect on the emulsifying activity, which remained almost constant, between 57.5 and 59.0% (data not shown). Bioemulsifiers that are not affected by NaCl concentrations up to 200–300 g/l have been described in bacteria [9], yeasts [12,13] and filamentous fungi [16]. However, in other cases, the emulsifying activity was significantly inhibited at NaCl concentrations higher than 50–100 g/l [4,11,23,25]. Also, the common chemical surfactants SDS (sodium dodecyl sulfate), Triton X-100 or Tween 80 do not show emulsifying activity at NaCl concentrations of 100–120 g/l [11,13].

The pH stability of the bioemulsifier was also evaluated. Bioemulsifier solutions (1 g/l) were prepared at different pH values (from 2 to 13) and the emulsifying values were measured. The emulsifying activity was almost constant (56.2-59.7%) over the entire range of pH values studied (data not shown). Several bioemulsifiers exhibit higher emulsifying activity at acidic [16] or alkaline conditions [4,9,25]. In other cases, they are only stable at neutral pH values [23,27]. Bioemulsifiers produced by some yeasts are also stable in a broad spectrum of pH values (from 2 to 10) [12,13,19,20]. Alasan and the bioemulsifier produced by *Streptomyces* sp. MC1 are stable at pH values between 3 and 10 [3,20,26].

To evaluate the stability of the bioemulsifier at high temperatures, bioemulsifier solutions (1 g/l) were exposed to 100°C for 1 h, and to 121°C for 20 min. Emulsification activity was measured before and after heating, and it was found that the heat treatment did not reduce the emulsion forming capacity of the bioemulsifier against *n*-hexadecane (data not shown). Different bioemulsifiers were shown to remain stable after exposure to 100°C for 1 h [8,12,13] or to 121°C for 20 min [6,23]. However, other bioemulsifiers lose some emulsifying capacity at those temperatures [4,9,16,25,27]. An exception is alasan, produced by *Acinetobacter radioresistens* KA53. The activity of this bioemulsifier increases approximately 30% after incubation at 100°C for 10 min, which is accompanied by changes in the conformation of the polysaccharide-protein complex [26].

The high stability of the bioemulsifier herein produced by *Paenibacillus* sp. #510 regarding temperature, pH and salinity clearly demonstrates its potential for applications involving extreme environmental conditions.

### Emulsifying activity using different hydrophobic substrates

The ability to emulsify different hydrocarbons was investigated using a wide range of pure and mixed substrates (Table 2). The bioemulsifier formed stable emulsions with aliphatic (*n*-hexadecane, *n*-hexane, chloroform, dichloromethane, ethyl acetate) and aromatic (toluene, xylene) hydrocarbons, as well as with hydrocarbon mixtures (crude oil, heating oil, paraffin). Gas oil was the only hydrocarbon not efficiently emulsified, whereas crude oil was the best substrate. A broad-spectrum of emulsifying activity is essential for the use of a bioemulsifier in industrial processes, such as bioremediation, treatment of industrial effluents, oil tank clean-up, emulsion-facilitated oil transport or emulsion-based fuels, because those processes include different mixtures of hydrophobic compounds. The results herein obtained show that the bioemulsifier produced by *Paenibacillus* sp. #510 possesses a high ability to stabilize emulsions with different hydrocarbons, thus representing a potential candidate to be used in a variety of biotechnological and industrial applications. The ability of bioemulsifiers to stabilize emulsions with hydrocarbons of different nature has been described by several authors [3,5,6,11,12,14,23]. However, the other bioemulsifiers reported exhibit considerable substrate specificity. Emulsan does not emulsify pure aliphatic, aromatic or cyclic hydrocarbons; however, mixtures of those compounds can be efficiently emulsified [3]. In other cases, pure hydrocarbons are efficiently emulsified, but the same is not seen for their mixtures [9].

**Table 2:** **Emulsifying indexes obtained with different hydrocarbons using the bioemulsifier produced by** ***Paenibacillus*** **sp. #510**

**Hydrocarbon**	**E**_**24**_ **(%)**
**Chloroform**	63.8 ± 0.7
**Crude oil**	75.1 ± 1.6
**Dichloromethane**	66.1 ± 1.9
**Ethyl acetate**	52.7 ± 1.3
**Gas oil**	15.9 ± 1.2
**Heating oil**	62.7 ± 1.5
***n*** **-hexadecane**	59.3 ± 1.0
***n*** **-hexane**	50.9 ± 1.8
**Paraffin**	63.1 ± 1.6
**Toluene**	61.6 ± 1.1
**Xylene**	59.3 ± 1.0

Results are expressed as means ± standard deviations of three independent experiments.

### Toxicity tests

The toxicity of the bioemulsifier produced by *Paenibacillus* sp. #510 and three chemical SACs was assessed using *Vibrio fisheri* as an indicator microorganism. The EC_50_ values obtained are shown in Table 3. Regarding the bioemulsifier, the results obtained didn’t allow calculating the EC_50_ value. After 30 min of exposure to the highest bioemulsifier concentration tested (1000 mg/l), the bioluminescence was reduced by 29%, showing that the bioemulsifier exhibits a low toxicity against *V. fisheri* as compared to the synthetic SACs studied. Furthermore, the results showed that this bioemulsifier exhibits equal or less toxicity when compared with other microbial SACs. In similar assays, Ivshina and co-workers [28] reported EC_50_ values for SACs produced by *Rhodococcus* spp. and *Pseudomonas aeruginosa* strains between 50 and 650 mg/l. Moreover, Lima et al. [29] reported EC_20_ values for different microbial SACs (including lipopeptides, glycolipids and flavolipids) between 261 and 736 mg/l, higher than the obtained for SDS, 25 mg/l. Franzetti and collaborators [8] also reported a low toxicity for the bioemulsifier produced by *Variovorax paradoxus* 7bCT5 against *V. fisheri*, with an inhibition of 34 ± 2% after 15 min of exposure to the highest concentration tested (500 mg/l).

**Table 3:** **Toxicity data obtained for the different SACs studied**

**Compound**	**EC**_**50**_ **(mg/l)**
**Glucopone®650**	4.9 ± 1.3
**Findet®1214 N/23**	9.6 ± 6.2
**LAS**	21.1 ± 4.8
**Bioemulsifier**	N.D.

Effective concentration (EC_50_) values obtained against *Vibrio fisheri* (after 30 min of exposure) for the three chemical SACs and the bioemulsifier produced by *Paenibacillus* sp. #510. Results are expressed as means ± standard deviations of three independent experiments. ND: not determined.

### Biodegradability tests

Biodegradability is an important feature when evaluating the environmental risk associated with the use of SACs. Microbial SACs are generally considered to be less toxic and more biodegradable than synthetic SACs. However, these properties are sometimes assumed as a direct consequence of their natural origin without further studies, and data available in the literature on their biodegradation is scarce [8,30].

The biodegradability of the chemical SACs and the bioemulsifier produced by *Paenibacillus* sp. #510 was assessed by liquid respirometric assays. COD and BOD provide information on the amount of oxygen necessary to degrade a compound through chemical or biological pathways, respectively.

COD values determined for Glucopone®650, Findet®1214 N/23 and LAS were 335.5 ± 0.5, 384.5 ± 3.5 and 345.5 ± 0.5 mg O_2_/l, respectively; in contrast with the value obtained for the bioemulsifier, 10.9 ± 0.2 mg O_2_/l. Regarding the BOD_5_, for the three chemical surfactants, the value obtained was around 35 mg O_2_/l, whereas in the case of the bioemulsifier that value was 9.5 ± 2.0 mg O_2_/l. The lower COD and BOD_5_ values obtained for this bioemulsifier when compared with the chemical SACs indicate that it is more easily biodegradable.

The ratio BOD_5_/COD gives an indication of the biodegradability of a given compound. Ratios higher than 0.4-0.6 suggest that the compound can be easily degraded by microorganisms. For the chemical SACs tested, the ratios obtained were around 0.1, thus demonstrating that they have a low biodegradability. Regarding the bioemulsifier, the ratio obtained (0.9) indicates an easier biodegradation.

Franzetti et al. [8] reported BOD_5_/COD ratios for the bioemulsifier produced by *V. paradoxus* 7bCT5 between 0.24 and 0.30, showing a moderate biodegradability by soil bacteria. Lima and co-workers [30] reported a higher biodegradability of different biosurfactants (including glycolipids, lipopeptides and flavolipids) when compared with the chemical surfactant SDS using pure and mixed bacterial cultures. Also, Mohan et al. [31] reported a higher biodegradability of rhamnolipids comparing with Triton X-100. The degradation of rhamnolipids by a bacterial consortium isolated from a soil sample under aerobic and nitrate-reducing conditions was also reported by Chrzanowski et al. [32]. Frank and co-workers [33] showed that a bacterial inoculum obtained from a soil sample was able to use different SACs (rhamnolipids, sophorolipids, trehalose tetraester and Tween 80) as sole carbon and energy source, but only rhamnolipids were completely degraded, whereas Tween 80 simply suffered a primary degradation. Biodegradability and toxicity are important features when evaluating the environmental risk associated with the use of SACs. It is often assumed that microbial SACs are more suitable for different applications than the synthetic surfactants due to their lower toxicity and higher biodegradability. Regarding the bioemulsifier produced by *Paenibacillus* sp. #510, toxicity data coupled with BOD_5_/COD ratios indicate that it is a greener option when compared with the synthetic surfactants studied.

### Preliminary chemical characterization

#### FT-IR analysis

**Figure 3:**
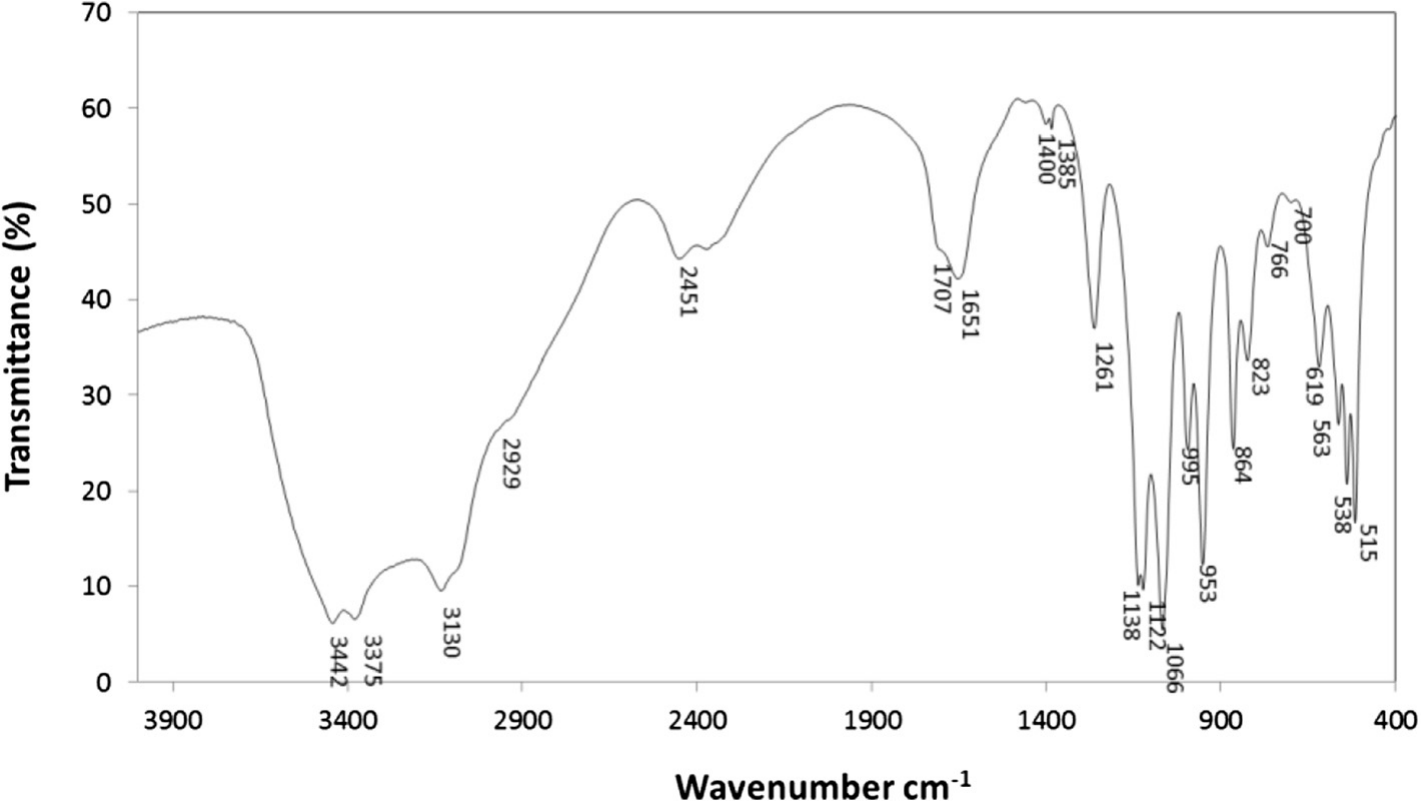
**FT-IR spectrum for bioemulsifier extract produced by** ***Paenibacillus*** **sp. #510.**

Firstly, a rough characterization of the bioemulsifier was performed by FT-IR analysis (Figure 3). The spectrum obtained clearly showed the characteristic bands belonging to carbohydrates, specifically O-H stretching at ca. 3400 cm^−1^ (e.g. 3375 and 3442 cm^−1^); and a set of intense bands between 1138 and 1000 cm^−1^ (1138, 1122 and 1066 cm^−1^) assigned to C-O-C anti-symmetric bridge stretching [34,35]. The peak at 1066 cm^−1^ can also be related to the C-OH stretching and the peak at 1122 cm^−1^ to primary C-OH group at the C6 position. On the other hand, it was also observed the presence of some characteristic bands of fatty acids, namely the band at 2929 cm^−1^ assigned to C-H stretching; and the relatively intense band at 1707 cm^−1^ assigned to C═O in non-conjugated fatty acids. A stretching vibration of a carboxylate ion in eventual uronic moieties of carbohydrates or in fatty acids at ca. 1650 cm^−1^ was also detected.

#### SEC analysis

The SEC analysis of the water soluble fraction of the bioemulsifier showed fairly narrow molecular weight distribution with rather low average molecular weight (Mw) of ca. 1000 Da obtained using pullulan calibration standards. Hence, the bioemulsifier should contain mono-dispersed oligosaccharides. The preliminary qualitative sugars analysis carried out by acid methanolysis revealed the presence of glucose as the major constituent of water-soluble oligosaccharides. Further evidence for the presence of oligosaccharides in the composition of the bioemulsifier was obtained using ^13^C CP-MAS NMR and ^1^H NMR analysis.

#### ^13^C CP-MAS NMR and ^1^H NMR spectroscopy

**Figure 4:**
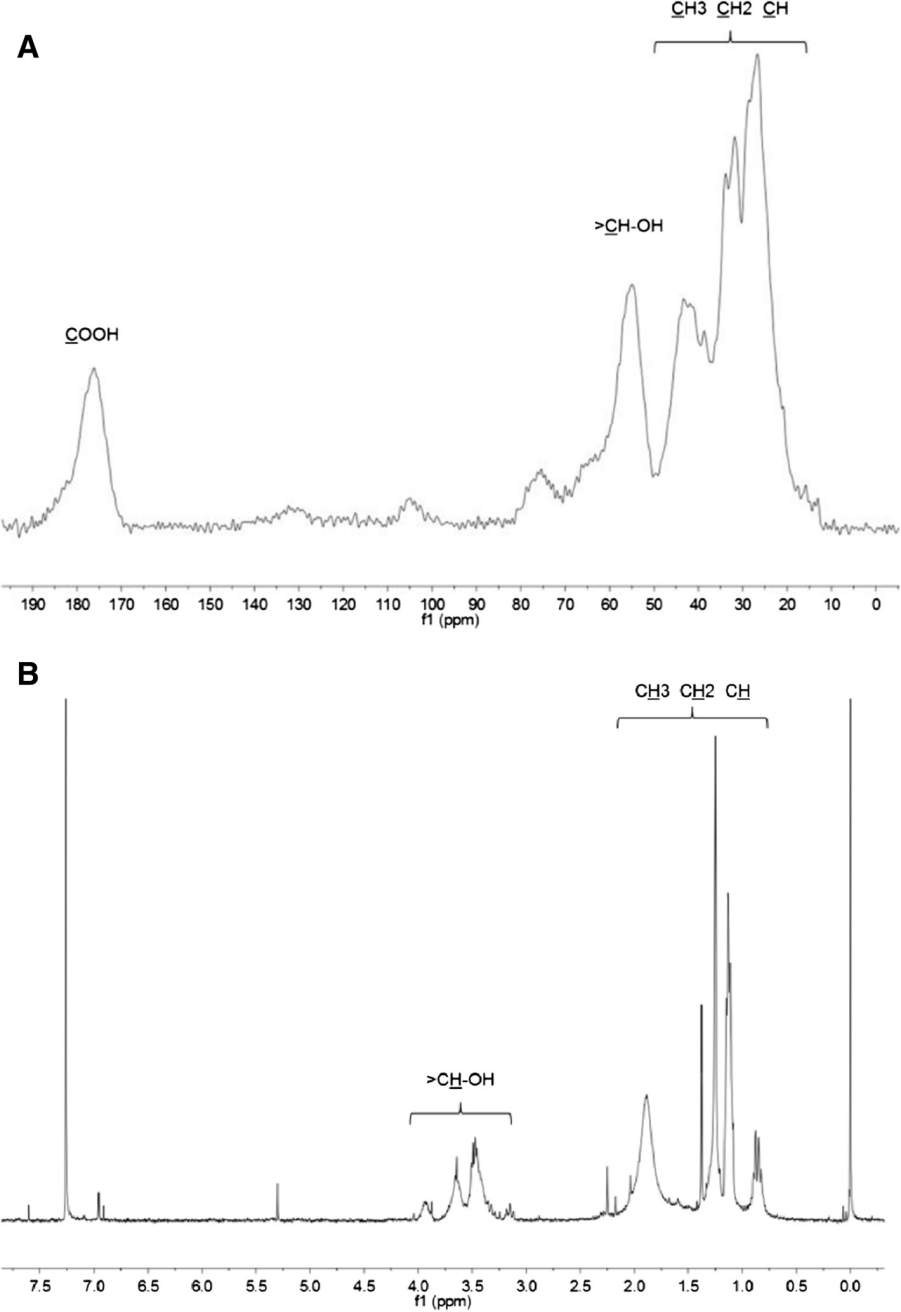
**Nuclear Magnetic Resonance spectra of the bioemulsifier produced by** ***Paenibacillus*** **sp. #510.** ^13^C CP-MAS NMR spectrum **(A)** and ^1^H NMR spectrum **(B)**.

^13^C CP-MAS NMR spectrum of the bioemulsifier suggests its eventual amphiphilic character. The strong resonances at 10–50 ppm assigned to aliphatic CH_3_/CH_2_/CH moieties, and a group of resonances centered at ca 176 ppm assigned to carbon of carboxylic groups [36,37], indicate an eventual major contribution of fatty acids (Figure 4A). At least part of these fatty acids should be hydroxylated as follows from the relatively intensive resonance at ca. 55 ppm assigned to the aliphatic carbon linked to the hydroxyl group. A series of resonances at 60–110 ppm may be assigned to oligosaccharides (Figure 4A). Thus, the resonances at 98–108 ppm are typical of glycosidic carbons (C1), and the resonances at 60–80 ppm of primary/secondary carbons (C2-C6) in pentosans and hexosans [37].

The presence of characteristic aliphatic and hydroxyl groups in the oligosaccharide-lipid complex was also confirmed by ^1^H NMR spectrum (Figure 4B). Noteworthy, only the chloroform-soluble counterpart of the bioemulsifier was analysed in this case. The ^1^H NMR spectrum of the bioemulsifier showed the presence of CH_3_, CH_2_, and CH groups as revealed from the resonances at 0.6-2.0 ppm, and the presence of > CH-OH moieties was evidenced from proton signals at 3.5-4.0 ppm [38]. At least part of the detected > CH-OH moieties may belong also to chemically bonded carbohydrates.

According to this preliminary chemical characterization, it seems that the bioemulsifier produced by *Paenibacillus* sp. #510 is a low molecular weight oligosaccharide-lipid complex in which the fatty acids and oligosaccharides could be structurally associated involving either covalent or non-covalent bonds. Therefore, further studies using advanced wet chemistry, NMR and mass spectrometry will be necessary to fully elucidate its structure.

Several species belonging to the genus *Acinetobacter* produce extracellular bioemulsifiers, with molecular weights around 1000 kDa. Emulsan, produced by *Acinetobacter venetianus* RAG-1, is a non-covalently linked complex of a lipoheteropolysaccharide and a protein. The polysaccharide (apo-emulsan) is composed mainly of D-galactosamine, D-galactosaminuronic acid and diamino-dideoxy glucosamine [2]. Surface activity of emulsan is largely due to the presence of long chain fatty acids, which are covalently linked to the polysaccharide backbone. The protein associated with the polysaccharide promotes the emulsifying activity [39]. Alasan consists of an anionic heteropolysaccharide containing covalently bound alanine and proteins [3]. The proteins are the active component of the emulsifier, and the purified polysaccharide (apo-alasan) shows very low emulsifying activity. Three proteins with molecular weights of 16, 31 and 45 KDa were isolated, which showed higher emulsifying activity compared to the apo-alasan, especially the 45 KDa protein, but the emulsion formed was considerably less stable than that produced by the entire molecule [26]. *Acinetobacter calcoaceticus* BD4 produces a bioemulsifier called BD4 emulsan. This strain produces a large anionic polysaccharide capsule composed of a repeating heptasaccharide unit containing L-rhamnose, D-glucose, D-glucuronic acid and D-mannose in molar ratios of 4:1:1:1. Under certain growth conditions, the capsule (hydrophilic) is released together with the bound protein (hydrophobic) into the medium, forming a complex with high emulsifying activity. The purified protein and the polysaccharide itself do not show emulsifying activity. However, the original emulsifying activity can be reconstituted by mixing both fractions [40]. Furthermore, as previously mentioned different bacteria, yeasts and filamentous fungi produce extracellular bioemulsifiers with different compositions (Table 4). However, all these compounds are high molecular weight bioemulsifiers, in contrast with the bioemulsifier produced by *Paenibacillus* sp. #510 herein studied.

**Table 4:** **Composition of bioemulsifiers produced by different bacteria, yeasts and filamentous fungi**

	**Composition**	**Microorganism**
**Bacteria**	C + L + P	*Aeribacillus pallidus* YM-1 [10]
		*Geobacillus pallidus* XS2 [9]
		*Lactobacillus pentosus* CECT4023 [41]
		*Variovorax paradoxus* 7bCT5 [8]
	C + L	*Aeromonas* spp. [4]
		*Rhodococcus* sp. TA6 [23]
	C + P	*Bacillus licheniformis* ACO1 [6]
		*Streptomyces* sp. MC1 [20]
	L + P	*Pseudomonas nitroreducens* TSB.MJ10 [11]
	C	*Halomonas eurihalina* F2-7 [5]
**Yeasts**	C + L + P	*Yarrowia lipolytica* [15]
	C + L	*Geotrichum* sp. [13]
		*Trichosporon* spp. [12,14]
**Filamentous fungi**	C + L + P	*Penicillium* sp. [16]

C: carbohydrate; L: lipid; P: protein.

## Conclusions

In this study, the production of a bioemulsifier by a novel *Paenibacillus* strain isolated from crude oil was reported. The preliminary chemical characterization revealed that it is a low molecular weight oligosaccharide-lipid complex. To our knowledge, the production of a low molecular weight bioemulsifier by a *Paenibacillus* strain has not been previously reported. The bioemulsifier was found to form stable emulsions with a variety of hydrophobic compounds and was not affected by exposure to extreme environmental conditions. Furthermore, the bioemulsifier exhibited a good environmental compatibility as can be concluded from the toxicity and biodegradability results obtained. Altogether, the features of this novel bioemulsifier make it an interesting biotechnological product for many environmental and industrial applications.

## Methods

### Characterization of the bioemulsifier producing strain

A promising bioemulsifier-producing strain (isolate #510) was isolated from a crude oil sample obtained from a Brazilian oil field and was identified by 16S rRNA sequencing. The 16S rRNA gene was amplified by PCR using primers fD1 and rD1. The resulting sequence (1451 bp) was compared with sequences in the GenBank database of the National Center for Biotechnology Information (NCBI) (http://www.ncbi.nlm.nih.gov) using the nucleotide-nucleotide blast (BLASTn) network service. Multiple sequence alignments were carried out using ClustalW and a consensus neighbor-joining tree was designed using Molecular Evolutionary Genetics Analysis (MEGA) Software version 5.1. The bioemulsifier production and characterization were performed according to the methodologies described in the following sections.

### Effect of culture conditions and hydrocarbons on bioemulsifier production

MSS medium was used to study the bioemulsifier production. The composition of MSS medium was (g/l): NaCl 10.0; sucrose 10.0; Na_2_HPO_4_ 5.0; NH_4_NO_3_ 2.0; KH_2_PO_4_ 2.0; MgSO_4_ · 7H_2_O 0.2. Flasks containing 50 ml of MSS medium were inoculated with a single colony from an agar plate. The effect of aeration on the bioemulsifier production was studied by incubating the cultures at 40°C under aerobic and anaerobic conditions at 120 rpm. Anaerobic cultures were prepared removing oxygen by aseptically bubbling oxygen-free nitrogen into the flasks, which were sealed with rubber stoppers. The ability of the strain to use hydrocarbons as the sole carbon source and the inductive effect of their presence on the bioemulsifier production were tested by growing the isolate in MSS medium with and without sucrose containing 5% (w/v) of paraffin or crude oil. The paraffin used was viscous paraffin, purchased from Merck (Merck, Darmstadt, Germany). Crude oil was obtained from a Brazilian oil field [42].

Samples were taken at different time points during the fermentation to determine the biomass concentration and bioemulsifier production. Bacterial growth was determined by measuring the optical density at 600 nm. Afterwards, the samples were centrifuged (10,000 × *g,* 20 min, 20°C) and cell-free supernatants were used to measure the surface tension and to determine the emulsifying activity, as described below. At the end of the fermentation, cells were harvested by centrifugation and the cell dry weight (g/l) was determined (48 h at 105°C). To recover the bioemulsifier, the cell-free supernatants were mixed with three volumes of cold ethanol and incubated at −20°C overnight. Afterwards, the precipitate was collected by centrifugation (10,000 × *g*, 20 min, 4°C). The crude bioemulsifier was dissolved in a minimal amount of demineralized water and freeze dried. The product obtained was weighed and stored at −20°C for further use.

### Surface-activity determination

Surface tension measurements of culture broth supernatants were performed according to the Ring method described elsewhere [43]. A KRÜSS K6 Tensiometer (KRÜSS GmbH, Hamburg, Germany) equipped with a 1.9 cm De Noüy platinum ring was used. To increase the accuracy of the surface tension measurements, an average of triplicates was conducted. All the measurements were performed at room temperature (20°C).

### Emulsifying activity determination

Emulsifying activity was determined by the addition of 2 ml of *n*-hexadecane to the same volume of cell-free culture broth supernatants or bioemulsifier solutions in glass test tubes. The tubes were mixed with a vortex at high speed for 2 min and then were incubated at 40°C for 24 h. The stability of the emulsion was determined after 24 h, and the emulsification index (E_24_) was calculated as the percentage of the height of the emulsified layer (mm) divided by the total height of the liquid column (mm). In order to study the ability of the bioemulsifier to form stable emulsions with different hydrophobic substrates, *n*-hexadecane was replaced in the emulsification assays by the following compounds: chloroform, crude oil, dichloromethane, ethyl acetate, gas oil, heating oil, *n*-hexane, liquid paraffin, toluene and xylene. All emulsification indexes were performed in triplicate.

### Effect of salinity, pH and temperature on bioemulsifier activity

The effect of several environmental parameters on the activity of the bioemulsifier produced by the microbial isolate was determined. Stability studies were performed using bioemulsifier solutions prepared in distilled water at a concentration of 1 g/l. In order to assess the effect of salinity on the bioemulsifier activity, bioemulsifier solutions were supplemented with different NaCl concentrations (from 10 to 300 g/l) and the emulsifying activity was measured as described above. To evaluate the stability of the bioemulsifier at high temperatures, bioemulsifier solutions were incubated at 100°C for 1 h and at 121°C for 20 min; the samples were then cooled to room temperature and the emulsification indexes were measured and compared to the corresponding values before heat treatment. The pH stability was studied by adjusting the bioemulsifier solutions to different pH values (2–13) using HCl or NaOH solutions, and then the emulsifying activity was measured as previously described. All the experiments were carried out in triplicate.

### Toxicity tests

Toxicity tests were performed using the bioemulsifier produced by the isolate #510 and three chemical SACs, namely Glucopone®650 (Fluka, Sigma-Aldrich), Findet®1214 N/23 (Kao Corporation, Tokyo, Japan) and LAS (Kao Corporation, Tokyo, Japan). Microtox® assays were carried out according to the manufacturer’s instructions. A 1000 mg/l starting solution (in distilled water) was prepared for each compound. Bioluminescence was measured in the Microtox® M500 analyzer. The toxicity of the different SACs on the bioluminescent bacterium *V. fisheri* was evaluated by measuring the reduction of light emission by this microorganism when exposed to different concentrations of SACs for 30 min as compared to the control (distilled water). Whenever possible, the EC_50_ (effective concentration of the test substance that caused a 50% reduction in the amount of luminescence emitted by the bacterial suspension after 30 min of exposure) was calculated.

### Biodegradability tests

The biodegradability of the bioemulsifier produced by the isolate #510 and the three chemical SACs in liquid medium was determined using respirometric tests. The Biochemical Oxygen Demand (BOD) was performed using 500 ml respirometric bottles (OxiTop®Respirometer System) which were filled with 250 ml of the bioemulsifier (1000 mg/l) or the chemical SACs (200 mg/l) solutions along with 2 ml of a soil bacteria inoculum (OD_600_ = 1) obtained by adding 0.5 g of a soil sample to 50 ml of rich medium. A control bottle (without SACs) was assembled to measure the basal activity. Bottles were incubated at 20°C for 5 days, and BOD_5_ was calculated by the difference between oxygen consumption in each bottle and the control. The Chemical Oxygen Demand (COD) was measured using a kit (Hach Company, USA) following the manufacturer’s instructions.

### Bioemulsifier chemical characterization

#### Extraction and purification

The bioemulsifier was extracted from cell-free supernatants using the Folch extraction method that is commonly used to extract lipids from biomolecules. The Folch extraction procedure was performed as described elsewhere [44]. Briefly, a chloroform/methanol mixture (2:1) was added to the supernatant sample to a final chloroform/methanol/water ratio of 8:4:3. The mixture was centrifuged (9000 × *g*, 5 min), the organic layer was collected and the samples were evaporated to dryness under N_2_ at 37°C for 30 min. Prior to ^1^H NMR spectroscopy analysis, the samples were re-dissolved in deuterated chloroform (CDCl_3_).

#### Fourier transform infrared spectroscopy (FT-IR) analysis

The bioemulsifier recovered from the cell free supernatant was characterized by FT-IR. To perform the FT-IR assays, pellets were prepared by mixing 2 mg of biosurfactant with 100 mg of potassium bromide and pressing them at 9 metric tons for 3 min. The pellet of biosurfactant was inserted in the FT-IR spectrometer and the respective infrared spectrum obtained. FT-IR spectra were collected on a Bruker Tensor 27 FT-IR spectrometer, using KBr pellets. Data were recorded at room temperature, in the range 4000 to 500 cm^−1^ by accumulating 64 scans with a resolution of 4 cm^−1^.

#### Gel permeation chromatography (GPC) and sugars analysis

The GPC analysis of the bioemulsifier, dissolved in ultra-pure water (HPLC for gradient analysis, ACROS Organics Chem. Co.) containing 0.1 M NaNO_3_ and 0.02% NaN_3_ to a concentration of ca. 1.0%, was carried out on a PL-GPC 110 system (Polymer Laboratories Ldt., UK) equipped with refraction index (RI) detector using two Plaquagel-OH MIXED 8 μm columns (300 mm × 7.5 mm) protected by Plaquagel 8 μm guard pre-column (Polymer Laboratories Ltd., UK). The temperature of the injector and the columns was kept constant at 36°C. The eluent, ultra-pure water containing 0.1 M NaNO_3_ and 0.02% NaN_3_, was pumped at a flow rate of 0.9 ml/min. The SEC columns were calibrated using pullulan standards (Polymer Laboratories Ldt., UK). The preliminary analysis on the sugar composition by acid methanolysis was carried out according to a previously published procedure [45].

#### Nuclear Magnetic Resonance (NMR) spectroscopy

^13^C solid-state Cross Polarization-Magic Angle Spinning Nuclear Magnetic Resonance (^13^C CP-MAS NMR) spectra were recorded on a BrukerAvance400 spectrometer operating at 100.6 MHz (9.4 T). Samples were spun in a zirconia’s Bruker rotor at 7 kHz. Acquisition parameters were as follows: 90° proton pulse of 4 μs width, contact time 2 ms, and pulse delay of 4 s. The ^1^H NMR spectra of bioemulsifier soluble in CDCl_3_ were recorded at 25°C on a BrukerAvance 300 spectrometer operating at 300.13 MHz. Radio frequency was 90° pulse, width of 10.2 μs, relaxation delay 12 s, and 200–400 scans. The chemical shifts are reported relative to tetramethylsilane (TMS) used as an internal standard (δ = 0.00).
